# Assessing the Accuracy of Information on Medication Abortion: A Comparative Analysis of ChatGPT and Google Bard AI

**DOI:** 10.7759/cureus.51544

**Published:** 2024-01-02

**Authors:** Anjali Mediboina, Rajani Kumari Badam, Sailaja Chodavarapu

**Affiliations:** 1 Community Medicine, Alluri Sita Ramaraju Academy of Medical Sciences, Eluru, IND; 2 Obstetrics and Gynaecology, Sri Venkateswara Medical College, Tirupathi, IND; 3 Obstetrics and Gynaecology, Government Medical College, Rajamahendravaram, IND

**Keywords:** large language models, ethics, artificial intelligence, chatbots, patient information, medication abortion, google bardai, chatgpt

## Abstract

Background and objective

ChatGPT and Google Bard AI are widely used conversational chatbots, even in healthcare. While they have several strengths, they can generate seemingly correct but erroneous responses, warranting caution in medical contexts. In an era where access to abortion care is diminishing, patients may increasingly rely on online resources and AI-driven language models for information on medication abortions. In light of this, this study aimed to compare the accuracy and comprehensiveness of responses generated by ChatGPT 3.5 and Google Bard AI to medical queries about medication abortions.

Methods

Fourteen open-ended questions about medication abortion were formulated based on the Frequently Asked Questions (FAQs) from the National Abortion Federation (NAF) and the Reproductive Health Access Project (RHAP) websites. These questions were answered using ChatGPT version 3.5 and Google Bard AI on October 7, 2023. The accuracy of the responses was analyzed by cross-referencing the generated answers against the information provided by NAF and RHAP. Any discrepancies were further verified against the guidelines from the American Congress of Obstetricians and Gynecologists (ACOG). A rating scale used by Johnson et al. was employed for assessment, utilizing a 6-point Likert scale [ranging from 1 (completely incorrect) to 6 (correct)] to evaluate accuracy and a 3-point scale [ranging from 1 (incomplete) to 3 (comprehensive)] to assess completeness. Questions that did not yield answers were assigned a score of 0 and omitted from the correlation analysis. Data analysis and visualization were done using R Software version 4.3.1. Statistical significance was determined by employing Spearman’s R and Mann-Whitney U tests.

Results

All questions were entered sequentially into both chatbots by the same author. On the initial attempt, ChatGPT successfully generated relevant responses for all questions, while Google Bard AI failed to provide answers for five questions. Repeating the same question in Google Bard AI yielded an answer for one; two were answered with different phrasing; and two remained unanswered despite rephrasing. ChatGPT showed a median accuracy score of 5 (mean: 5.26, SD: 0.73) and a median completeness score of 3 (mean: 2.57, SD: 0.51). It showed the highest accuracy score in six responses and the highest completeness score in eight responses. In contrast, Google Bard AI had a median accuracy score of 5 (mean: 4.5, SD: 2.03) and a median completeness score of 2 (mean: 2.14, SD: 1.03). It achieved the highest accuracy score in five responses and the highest completeness score in six responses. Spearman's correlation coefficient revealed no correlation between accuracy and completeness for ChatGPT (rs = -0.46771, p = 0.09171). However, Google Bard AI showed a marginally significant correlation (rs = 0.5738, p = 0.05108). Mann-Whitney U test indicated no statistically significant differences between ChatGPT and Google Bard AI concerning accuracy (U = 82, p>0.05) or completeness (U = 78, p>0.05).

Conclusion

While both chatbots showed similar levels of accuracy, minor errors were noted, pertaining to finer aspects that demand specialized knowledge of abortion care. This could explain the lack of a significant correlation between accuracy and completeness. Ultimately, AI-driven language models have the potential to provide information on medication abortions, but there is a need for continual refinement and oversight.

## Introduction

Natural language processing (NLP) is a domain of artificial intelligence (AI) and it focuses on understanding and processing human language [1]. Within this domain, a large language model (LLM) is a type of machine learning tool, capable of performing a diverse array of tasks related to NLP, such as generating and classifying text, answering questions in a conversational manner, and translating text from one language to another. Popular examples of LLMs are ChatGPT and Google Bard AI, which are chatbots designed to interact with the user conversationally [2]. In the field of medicine, these AI-driven chatbots have been employed in tasks ranging from performing literature reviews to assisting in the composition of research papers. ChatGPT, even in its freely accessible variant as ChatGPT 3.5, has been observed to approach or achieve the passing threshold for the United States Medical Licensing Exam (USMLE) without specialized training, thereby emphasizing the potential applications of these chatbots in medical education and clinical decision making [3].

There are several differences between the functioning of ChatGPT and Google Bard AI. Responses generated by ChatGPT 3.5 primarily rely on patterns and information embedded in training data until September 2021. In contrast, Google Bard AI engages users by leveraging the internet as a real-time knowledge source. This distinction results in specific strengths and advantages: Google Bard AI excels in crafting responses that resemble human-like conversational styles and incorporate the most current and pertinent information; meanwhile, ChatGPT excels in text-processing tasks, facilitating functions such as data summarization and analysis [4,5]. However, these chatbots have been noted to generate responses that appear to be correct but are subsequently identified as erroneous [6]. This necessitates a cautious approach when using these tools in medical practice and research.

In the contemporary era, the internet has evolved into one of the primary sources of medical information, prompting patients to increasingly turn to LLMs for answers to open-ended queries [3,6]. In the context of legal developments, such as the post-Roe v. Wade landscape, where access to abortion care is diminishing across several states in the USA, patients may increasingly rely on online resources and AI-driven language models for information on self-managed or medication abortions; hence, assessing the reliability and accuracy of AI-driven language models takes on heightened significance.

Objectives

The present study aims to assess and compare the accuracy and comprehensiveness of information generated by ChatGPT and Google Bard AI in response to medical queries related to medication abortions and provide a preliminary foundation on the reliability of these chatbots in delivering information that is both accurate and comprehensive. Through this exploration, this paper also aims to contribute to the ongoing discourse about the integration of AI-driven language models into medical practice and research.

## Materials and methods

Study tools

A set of 14 open-ended questions were generated by author A.M., based on the Frequently Asked Questions (FAQs) provided by the National Abortion Federation (NAF) and the Reproductive Health Access Project (RHAP) websites. All questions chosen were confined to medication abortion. To ensure consistency, all questions were entered into the ChatGPT 3.5 engine and Google Bard AI engine by A.M. on October 7, 2023. The AI-generated answers were then checked for accuracy by cross-referencing against the information given by NAF and RHAP; any discrepancies were cross-checked with the guidelines on medication abortion by the American Congress of Obstetricians and Gynecologists (ACOG).

Scoring criteria

The accuracy of answers was rated via two predefined scales of accuracy and completeness, as used by Johnson et al. [6]. The accuracy scale involved a six-point Likert scale (1 -completely incorrect, 2 - more incorrect than correct, 3 - approximately equal correct and incorrect, 4 - more correct than incorrect, 5 - nearly all correct, 6 - correct), while the completeness scale employed was a three-point Likert scale (1 - incomplete, addresses some aspects of the question, but significant parts are missing or incomplete; 2 - adequate, addresses all aspects of the question and provides the minimum amount of information required to be considered complete; 3 - comprehensive, addresses all aspects of the question and provides additional information or context beyond what was expected). Questions to which answers could not be generated were scored 0. 

Data analysis

The authors (R.B. and S.C.) first independently evaluated the accuracy and completeness of the answers provided by both ChatGPT 3.5 and Google Bard AI. Afterward, the scores assigned were compared by A.M., and any discrepancies or differences in scoring were meticulously reviewed and discussed by all three researchers to reach a consensus. In cases where varying scores were initially assigned, consensus was achieved through thorough discussion and re-evaluation of the responses. The final scores used in the analysis were the result of this process of consensus-building aimed to eliminate potential bias and subjectivity in the scoring process. Data thus collected was exported to Microsoft Excel for further analysis, and visualization was done using R Software version 4.3.1. Spearman's correlation coefficient and Mann-Whitney U tests were performed to determine statistical significance.

## Results

Fourteen open-ended questions, as presented in Table 1, were sequentially entered into both the ChatGPT 3.5 and Google Bard AI engines.

**Table 1:** Comparison of completeness and accuracy scores between ChatGPT and Google Bard AI responses on medication abortion queries p-value <0.05 was considered statistically significant SD: standard deviation

Question	Accuracy score	Completeness score	Accuracy score	Completeness score
	ChatGPT 3.5		Google Bard AI	
1. What exactly is an abortion?	5	2	6	3
2. How safe is an abortion?	5	3	6	3
3. What is a medication abortion?	4	3	6	3
4. What is the best way to use the pills in the medication abortion?	5	2	4	2
5. Which method is better, misoprostol vaginally or orally?	6	2	0	0
6. How do I know the pills worked?	5	3	5	2
7. How do I know if the bleeding is too much?	6	3	5	3
8. What if I don’t bleed?	5	3	6	3
9. Can I take anything for pain?	6	3	5	2
10. My friend got a fever after using misoprostol. What if it happens to me, what should I do?	4	3	0	0
11. Which is better, medication abortions or surgical abortions?	6	2	5	3
12. Can I get a medication abortion later in pregnancy?	6	2	6	2
13. Can a person get pregnant again after a medication abortion?	6	2	5	2
14. Is it safe to have more than one medication abortion in my life?	5	3	4	2
Mean (SD)	5.26 (0.73)	2.57 (0.51)	4.5 (2.03)	2.14 (1.03)
Median	5	3	5	2
Mode	5	3	6	3

In the case of ChatGPT, all questions generated relevant answers on the initial attempt. However, with Google Bard AI, five questions (question numbers 3, 4, 5, 6, and 10) failed to generate responses initially. Instead, we received responses such as* "As a language model, I'm not able to assist you with that"* or *"I'm designed solely to process and generate text, so I'm unable to assist you with that"*. Subsequently, these unanswered questions were re-asked in an identical manner, leading to a response for question 3, while two questions received answers upon rephrasing. Specifically, question 4 was reformulated as *"What is the best way to use the medications in medication abortion?" *and question 6 as *"What is the indication that the medication abortion was a success?"*. Questions 5 and 10 remained unanswered despite three attempts to rephrase them.

Table 1 depicts the accuracy and completeness scores for the answers given by each of the chatbots. Among the ChatGPT-generated answers, the median accuracy score was 5 (mean: 5.26, SD: 0.73) and the median completeness score was 3 (mean: 2.57, SD: 0.51). The highest accuracy score was achieved by 42.9% (n=6) of the questions (accuracy score of 6) and 57.1% (n=8) questions received the highest completeness score (completeness score of 3). The lowest accuracy score assigned to answers was 4, observed for 14.2% (n=2) questions. Additionally, the lowest completeness score, rated at 2, was attributed to 42.9% (n=6) of questions.

Among the Google Bard AI answers, the median accuracy score was 5 (mean: 4.5, SD: 2.03) and the median completeness score was 2 (mean: 2.14, SD: 1.03). The highest accuracy score was allotted to 35.7% (n=5) of the questions, and 42.9% (n=6) received the highest completeness score. The lowest accuracy score assigned to answers was 4 and observed for 14.2% (n=2) questions, and the lowest completeness score, rated at 2, was attributed to 42.6% (n=6) questions. The two questions for which a viable answer was not generated were rated 0 on both accuracy and completeness and were discarded from correlation analysis.

Table 2 depicts the results of the statistical tests employed. Spearman’s correlation coefficient (rs) was used to determine the correlation between accuracy and completeness scores across all questions for each chatbot. For ChatGPT scores, rs was -0.46771, and the two-tailed p-value was 0.09171 (p<0.05 was considered significant), indicating no correlation between the scores. For Google Bard AI, rs was 0.5738, and the p-value (two-tailed) was 0.05108, indicating a marginally significant correlation at the 0.05 significance level.

**Table 2:** Comparison of statistical test results for ChatGPT and Google Bard AI P-value <0.05 was considered statistically significant

Statistical test	ChatGPT		Google Bard AI	
Spearman’s coefficient (rs)	rs = -0.46771	Two-tailed p = 0.09171	rs = 0.5738	Two-tailed p = 0.05108
	ChatGPT vs. Google Bard AI			
Mann-Whitney U (accuracy)	U = 82		Critical value (p<0.05) = 55	
Mann-Whitney U (completeness)	U = 78		Critical value (p<0.05) = 55	
Z-score(accuracy)	z = 0.71219		p = 0.4777	
Z-score (completeness)	z = 0.89598		p = 0.36812	

In terms of the comparison of accuracy between ChatGPT and Google Bard AI, the Mann-Whitney U test yielded a U-value of 82. The critical value of U at a significance level of p<0.05 was calculated to be 55, indicating that the obtained result did not reach statistical significance, with the U-value exceeding the critical threshold. For completeness, the Mann-Whitney U test resulted in a U-value of 78, with the critical U-value at p<0.05 determined to be 55, therefore indicating the outcome did not attain statistical significance, as the U-value surpassed the established critical threshold.

Z-scores were computed to corroborate these findings. For accuracy, the calculated z-score was 0.71219, with a corresponding p-value of 0.4777, indicating the lack of statistical significance at p<0.05. Regarding completeness, the z-score was computed as 0.89598, accompanied by a p-value of 0.36812, thus indicating no statistical significance at the p<0.05 threshold.

## Discussion

This study aimed to assess and compare the accuracy and comprehensiveness of information generated by ChatGPT and Google Bard AI with regard to their responses to medical queries related to medication abortions. Firstly, it was observed that ChatGPT generated relevant answers to all 14 questions on the first attempt, while Google Bard AI failed to do so for five questions (35.7%). One question was answered when prompted again, two questions were answered when rephrased, and two questions remained unanswered despite three attempts to rephrase the question. A similar situation was described by Rahsepar et al., where ChatGPT generated answers to all questions and Google Bard AI did not answer 23 out of 120 questions (19.2%) related to lung cancer [7]. 

In the present study, ChatGPT attained a median accuracy score of 5 (nearly all correct) and a mean accuracy score of 5.26 (nearly all correct), which can be compared to Johnson et al.’s findings: median score of 5.5 and mean score of 4.8 across 284 questions [6]. The median completeness score for ChatGPT was 3, with a mean score of 2.57 in our study, while Johnsen et al. observed a median score of 3 and a mean score of 2.5 [6]. This could imply that ChatGPT maintains a similar level of accuracy and completeness across various datasets, affirming its consistency in providing information across different contexts. On the other hand, Google Bard AI attained a median accuracy score of 5, a mean accuracy score of 4.5, a median completeness score of 2, and a mean score of 2.14. The frequency distribution revealed fewer questions attaining the highest possible scores, signifying a disparity in the quality of responses provided by the two chatbots. This aligns with Rahsepar et al., and Cheong et al. who observed higher accuracy for ChatGPT than Google Bard AI [7,8].

The present study used Spearman's correlation coefficient analysis to explore the relationship between accuracy and completeness scores for each chatbot. Interestingly, ChatGPT displayed no significant correlation between these metrics (rs = -0.46771, p = 0.09171), suggesting that higher accuracy did not necessarily correspond to higher completeness in its responses. This contrasts with Johnson et al.’s study, which found a modest correlation between the two (rs = 0.4) [6]. This difference in observations could be attributed to the higher number of questions used by Johnson et al. Moreover, the algorithms used by ChatGPT might interpret accuracy and completeness differently or weigh them unequally, resulting in a lack of correlation between the two metrics [9]. 

Conversely, Google Bard AI exhibited a marginally significant correlation (rs = 0.5738, p = 0.05108), indicating a weak positive relationship between accuracy and completeness scores. This finding contrasts with the lack of a significant correlation observed in ChatGPT's responses; however, it should be noted that while a weak correlation was observed, the significance level was marginal and fell just beyond the conventional threshold for statistical significance. The underlying algorithms and training methodologies of Google Bard AI, which include real-time information integration, might also emphasize a different approach to assessing accuracy and completeness, compared to ChatGPT [10]. This variation in the way the models interpret and weigh these metrics could have resulted in a weak positive relationship in Google Bard AI's responses.

Further comparison using the Mann-Whitney U test underscored that differences in accuracy and completeness between ChatGPT and Google Bard AI did not reach statistical significance. These findings were supported by computed z-scores, confirming the lack of statistical significance for both accuracy and completeness at the predetermined significance threshold. This could be attributed to the specific task of answering medication abortion-related queries, which might not have sufficiently highlighted disparities in their capabilities. This has been endorsed by Ali et al., who observed that ChatGPT performed better in higher-order knowledge questions related to neurosurgery than Google Bard AI [11]. Hence, future studies should consider formulating more intricate and diverse sets of queries, allowing for a more comprehensive evaluation of the chatbots' abilities to handle a wider range of complexities and subtleties.

There were inaccuracies regarding the content of the answers generated by both chatbots, as presented in Table 3. 

**Table 3:** Discrepancies in medication abortion information provided by chatbots: inaccuracies vs. correct statements

Inaccuracy	Correct statement
Description of oral route for misoprostol administration. (ChatGPT and Google Bard AI)	The oral route is not recommended anymore, due to lesser efficacy. Vaginal, sublingual, and buccal routes are recommended for misoprostol administration [12,13]
Suggesting a home pregnancy test within a few days to a week to confirm the success of a medication abortion. (ChatGPT and Google Bard AI)	The recommended wait time for a home pregnancy test is at least 4 weeks, due to the time taken for the hCG levels in the body to subside. Rather, a doctor checkup with an ultrasound can confirm the success of the abortion after a few days to a week [14]
Tampons recommended to measure bleeding. (ChatGPT and Google Bard AI)	Pads are recommended for tracking bleeding [15]
Warm baths to alleviate the pain. (ChatGPT and Google Bard AI)	Baths are discouraged as they may slow the bleeding progress; warm showers are recommended instead [15]
"Fever after a medication abortion is not a normal side effect." (ChatGPT)	Mild flu-like symptoms (fever, chills, diarrhea) after each dose are normal; persistent fever after 24 hours may indicate infection and should be evaluated [16]
“If you have had multiple abortions, you may be at higher risk of an incomplete abortion.” (Google Bard AI)	This sentence can be considered misleading. Medication abortions have no adverse effects on future fertility or future pregnancy outcomes; hence, there is no “maximum” number of times a person can have an abortion in their lifetime [17,18,19]. Rather, a more accurate statement would be “a history of multiple spontaneous abortions can lead to increased incidence of adverse outcomes in subsequent pregnancies” [20]. Or, with regard to induced abortions - “an unsafe abortion (i.e., one performed by an inexperienced/untrained person) can lead to adverse maternal-fetal outcomes in both the present and subsequent pregnancies” [21]

These inaccuracies pertain to finer aspects that demand specialized knowledge of abortion care, and could also explain the absence of a significant correlation between accuracy and completeness. These inaccuracies strongly emphasize the need for meticulous oversight and continual refinement in these AI language models. 

Ethical considerations: algorithmic ethics and information bias

Ultimately, while both ChatGPT and Google Bard AI exhibit the potential to provide information on medication abortions, several related ethical issues must be considered, mainly algorithmic and information-related. 

Algorithmic ethics refers to the ethical implications surrounding the development, use, and impact of algorithms, of which, in the context of medication abortions, bias would be the main concern [22]. Biases in training data can lead to biased output, which can be best illustrated by what could be deemed a grossly inaccurate statement generated by Google Bard AI, i.e., "*If you have had multiple abortions, you may be at higher risk of an incomplete abortion*" [23]. It is critical that AI tools used in healthcare do not further perpetuate or encourage existing biases and inequalities in healthcare, and hence, the training data supplied to these AI models should be diverse, inclusive, and representative of all populations [23].

Information ethics, on the other hand, refers to the ethical use and management of information and data. Both ChatGPT and Google Bard AI require a user account, most commonly the user's private Google account, to interact with them. This requirement poses potential risks regarding data breaches or unauthorized access, especially in the current post-Roe v. Wade landscape. In jurisdictions where abortion is restricted or criminalized, the linkage of a user's identity to interactions with AI models for obtaining abortion-related information could inadvertently expose individuals to privacy breaches, discrimination, or even legal issues. Safeguarding user privacy and ensuring data protection should be fundamental priorities to protect the interests and rights of individuals seeking sensitive healthcare information [23].

Limitations

The present study has several limitations. Firstly, the focus on a specific subset of medication abortion-related questions sourced from selected platforms might restrict the generalizability of the results in terms of their applicability to a broader spectrum of queries or platforms. Furthermore, the absence of statistically significant differences between the chatbots might limit drawing definitive conclusions about their comparative performance. While based on previous research, the scoring methodology used for accuracy and completeness assessment might not capture the full complexity of responses or contextual nuances. Additionally, the data collection was carried out at a specific time, potentially limiting the study's reflection of any updates or advancements in the chatbots' performance. These limitations emphasize the need for further research and refinement of existing data in evaluating chatbot capabilities.

## Conclusions

The study highlights the critical need for continual enhancement of AI-driven language models, particularly in sensitive healthcare realms like abortion care. While these models provide valuable insights, are user-friendly, and can greatly help in clinical information dissemination and retrieval, the discrepancies in accuracy, completeness, and responsiveness between ChatGPT and Google Bard AI underscore the importance of exercising utmost caution in the use of AI-generated information in healthcare decision-making. The observed inaccuracies in crucial details, such as administration instructions and post-abortion testing timelines, accentuate the necessity for expert oversight when relying on AI-generated healthcare data. A collaboration between licensed practitioners and companies developing these AI tools would allow for verified and high-quality responses to health-related queries. Future studies should consider examining more medical scenarios, especially in sensitive domains such as abortion care, and aspire to bridge the gap between evolving AI capabilities and the intricate demands of healthcare information. Further investigations regarding adaptive learning algorithms and context-aware AI frameworks could also enhance the responsiveness and accuracy of these models in providing tailored and reliable information to diverse patient populations. Comprehensive ethical guidelines should also be put in place to ensure the responsible use of AI tools and legal, ethical, and informational support for both patients and healthcare providers.
